# Optimal culture conditions for neurosphere formation and neuronal differentiation from human dental pulp stem cells

**DOI:** 10.1590/1678-7757-2021-0296

**Published:** 2021-10-01

**Authors:** Thanasup GONMANEE, Tawepong ARAYAPISIT, Kutkao VONGSAVAN, Chareerut PHRUKSANIYOM, Hathaitip SRITANAUDOMCHAI

**Affiliations:** 1 Mahidol University Faculty of Medicine Ramathibodi Hospital Chakri Naruebodindra Medical Institute Samut Prakan Thailand Mahidol University, Faculty of Medicine Ramathibodi Hospital, Chakri Naruebodindra Medical Institute, Samut Prakan, Thailand.; 2 Mahidol University Faculty of Dentistry Department of Anatomy Bangkok Thailand Mahidol University, Faculty of Dentistry, Department of Anatomy, Bangkok, Thailand.; 3 Walailak University International College of Dentistry Department of Pediatric Dentistry Bangkok Thailand Walailak University, International College of Dentistry, Department of Pediatric Dentistry, Bangkok, Thailand.; 4 Mahidol University Faculty of Dentistry Department of Pharmacology Bangkok Thailand Mahidol University, Faculty of Dentistry, Department of Pharmacology, Bangkok, Thailand.; 5 Mahidol University Faculty of Dentistry Department of Oral Biology Bangkok Thailand Mahidol University, Faculty of Dentistry, Department of Oral Biology, Bangkok, Thailand.

**Keywords:** Cell culture techniques, Mesenchymal stem cells, Neuronal differentiation, Progenitor cells

## Abstract

**Objectives:**

Human dental pulp stem cells (DPSCs) have been used to regenerate damaged nervous tissues. However, the methods of committing DPSCs into neural stem/progenitor cells (NSPCs) or neurospheres are highly diverse, resulting in many neuronal differentiation outcomes. This study aims to validate an optimal protocol for inducing DPSCs into neurospheres and neurons.

**Methodology:**

After isolation and characterization of mesenchymal stem cell identity, DPSCs were cultured in a NSPC induction medium and culture vessels. The durations of the culture, dissociation methods, and passage numbers of DPSCs were varied.

**Results:**

Neurosphere formation requires a special surface that inhibits cell attachment. Five-days was the most appropriate duration for generating proliferative neurospheres and they strongly expressed Nestin, an NSPC marker. Neurosphere reformation after being dissociated by the Accutase enzyme was significantly higher than other methods. Passage number of DPSCs did not affect neurosphere formation, but did influence neuronal differentiation. We found that the cells expressing a neuronal marker, β-tubulin III, and exhibiting neuronal morphology were significantly higher in the early passage of the DPSCs.

**Conclusion:**

These results suggest a guideline to obtain a high efficiency of neurospheres and neuronal differentiation from DPSCs for further study and neurodegeneration therapeutics.

## Introduction

Neurodegenerative disorders are characterized by irreversible progressive loss of neurons in the brain, which brings suffering to millions of people worldwide. Regeneration of the brain via endogenous neurogenesis is very limited and restricted to its region.^1,2^ Neural stem/progenitor cells (NSPCs), which are self-renewing, multipotent cells residing in the regenerative area of the brain, have been intensively studied for their potential use to regenerate damaged nervous tissue. During brain tissue repair, the endogenous NPSCs infiltrate into the lesion site to process the neurogenesis and replace the lost tissue. However, the brain fails to regenerate functional tissue due to the low incidence of NSPCs in adult humans and the lack of structural support for the migration of the NPSCs.^3^ Thus, transplantation of exogenous sources of stem cells with neuronal potential—combined with the presence of secretome, growth factors, and supportive extracellular matrix—has been suggested as a plausible approach for neurodegenerative disease treatment.^4,5,6^

Apart from the brain, NSPCs can be derived from various types of stem cells via culturing in a serum-free medium consisting of epidermal growth factor (EGF) and basic fibroblast growth factor (bFGF) that enable neural specification and maintain proliferative activity, followed by subsequent terminal differentiation into neurons or glial cells.^7,8^ NSPCs are usually cultured on a non-adhesive surface to form a free-floating clustering of cells known as a neurosphere.^7^ The neurosphere cultures also present an extracellular matrix typically enriched in the brain tissue and expresses synaptic and ion transport machinery.^9^Formation of the neurosphere depends on many factors already shown in several studies, such as passage number of initial cells,^10^ type of culture vessel surface,^11^ time of culture,^12^ and method of neurosphere dissociation.^13^ However, these factors have been investigated independently and each study used cells from different sources. Thus, the standard protocol that researchers should adopt for maximizing the neurosphere formation that contributes to the highest potential of neuronal differentiation has not yet been determined.

Human dental pulp stem cells (DPSCs) are mesenchymal stem cells located in a perivascular niche that play an essential role in dentin and pulp regeneration.^14,15^ During mastication or injury of the teeth, DPSCs respond to biochemical cues from extracellular matrix change, neighboring cells, and physical signals from mechanical stresses, resulting in proliferation, and both odontogenic and osteogenic differentiation.^16^ According to embryonic development, DPSCs originate from the ectomesenchyme, since they express neural crest cell markers,^17^ which indicate the same origin as the nervous system. This brought a lot of attention from researchers to the study of neuronal differentiation potential of cells as a regenerative source for neurodegenerative disease. Despite DPSCs having been differentiated into multiple types of neurons, the methods of NSPC induction are highly varied according to the desired terminal differentiation.^18^ The most noticeable variable is the duration of neurosphere formation, which could be from 4 to 15 days.^19,20^ The passage number of cells used for NSPC induction is also slightly different amongst studies; however, they are mostly considered to be of early passage.^21,22^ Nevertheless, there are no specific guidelines regarding optimal conditions for a high yielding NSPCs induction of DPSCs.

Our study aims to demonstrate a standard method to induce DPSCs into NSPCs and differentiate them into neuronal cells. We explored the factors involved in the neurosphere formation, including culture vessel surface, duration of the culture, methods to dissociate the neurospheres, and passage number of DPSCs. We also continued the study on the effect of DPSCs passage number on neuronal differentiation after the NPSC commitment. The efficiency of NSPC induction was determined by neurosphere size, number, and expression of neural progenitor markers; and the neuronal differentiation was evaluated by neuronal marker expression and morphology of the cells.

## Methodology

### Tooth collection

This study was approved by the Ethical Committee on Human Rights Related to Human Experimentation of the Faculty of Dentistry/Faculty of Pharmacy, (MU-DT/PY-IRB 2014/044.2710). Three healthy permanent teeth (n=3) were collected from Thai patients at the Faculty of Dentistry. Teeth with caries, restoration, history of trauma, and/or signs of pulpal pathology were excluded from this study.

### Isolation, maintenance, and characterization of human dental pulp stem cells

The human dental pulp stem cells (DPSCs) were isolated via outgrowth method, cultured, and characterized as described in our previous study.^23^ Briefly, pulp tissues were submerged in a cell culture medium containing Dulbecco’s modified Eagle’s medium (DMEM, HyClone, Fisher Scientific, Loughborough, UK), 10% fetal bovine serum (FBS, Biochrome, Berlin, GY), 100 U/mL Penicillin (Gibco, Thermo Fisher Scientific), and 100 μm/mL Streptomycin (Gibco), then maintained in a humidified atmosphere with 37°C and 5% CO_2_. The medium was changed every other day until the confluence of the outgrowth cells reached 80%, then they were sub-cultured or harvested using 0.05% Trypsin/EDTA (Gibco). Characterization of the cells was performed as follows: detection of mesenchymal stem cell surface markers, colony-forming unit fibroblast, and multipotential differentiation. The cells were incubated with antibodies against mesenchymal stem cell surface antigens (CD44, CD73, CD105, and CD146), hematopoietic stem cell antigen (CD34), and NSPC marker, Nestin (all from BioLegend, San Diego, CA, USA), through flow cytometry. Colony-forming unit fibroblast was performed by culturing the cells at a low density (500 cells/ml) for 2 weeks, after which the colonies were counted. Finally, multipotential differentiation was conducted by culture cells with the Advanced STEM Osteogenic Differentiation Medium Kit (GE Healthcare Technologies, West Milwaukee, WI, USA) and the Advanced STEM Adipogenic Differentiation Kit (GE Healthcare Technologies) for 28 days. Osteogenic and adipogenic differentiation were observed with Alizarin Red staining and Oil Red O staining, respectively.

### Neural stem/progenitor cell induction (Neurosphere assay)

DPSCs were cultured with 20,000 cells/cm^2^ density in Neural stem/progenitor cells (NSPC) medium containing DMEM/F12 (Gibco), 20 ng/mL bFGF (Gibco), 20 ng/mL EGF (Gibco), B-27 (Gibco), 100 U/mL Penicillin (Gibco), and 100 μm/mL Streptomycin (Gibco). Half of the medium was changed every other day. The cells were maintained in a humidified atmosphere with 37°C and 5% CO_2_. The culture vessels used in the culture were non-treated plates (JET BIOFIL, Guangzhou, CN) and Nunclon Sphera plates (Thermo Fisher Scientific). Durations of the culture were determined at day 0 to day 7.

### Neurosphere dissociation

Neurospheres were collected by centrifugation at 700 rpm for 5 min, then resuspended in 1 mL fresh NSPC medium. The dissociation was performed mechanically and enzymatically. Mechanical dissociation was performed by P1000 repeated pipetting for 100, 200, and 500 times. For enzymatic dissociation, Trypsin-EDTA (Gibco) and Accutase (Gibco) were used. After discarding the supernatant, the neurospheres were resuspended in either 1 mL 0.125% Trypsin-EDTA or 1 mL 1× Accutase. Neurospheres in Trypsin-EDTA were incubated for 3 min at 37°C and inactivated by adding 1 mL DPSCs culture medium. Neurospheres in Accutase were incubated for 10 min at room temperature. Subsequently, the volumes of neurosphere suspension in both enzymes were adjusted to 5 mL by adding the fresh NSPC medium. The dissociated NSPCs were collected by centrifugation at 3,000 rpm for 5 min, then the neurosphere assay was performed to determine the survival of the NSPCs.

### Neuronal differentiation of DPSCs-derived NSPC

DPSCs at passage 5, 10, and 15 were induced into NSPC by the neurosphere assay. Neurospheres derived from DPSCs of each passage number were dissociated and seeded onto poly-L-ornithine (Sigma, St. Louis, MO, USA) and laminin (Sigma) coated surface plates with 2×10^4^ cells/cm^2^ density. The cells were cultured in neuronal differentiation medium consisting of DMEM/F12, 20 ng/mL brain-derived neurotrophic factor (BDNF, Sigma), 20 ng/mL neurotrophin-3 (NT-3, Sigma), 20 ng/mL glial cell-derived neurotrophic factor (GDNF, Sigma), 2% N-2 (Gibco), B-27, 100 U/mL Penicillin, and 100 μm/mL Streptomycin. Dissociated neurospheres were cultured in the NSPC induction medium as a control group. The culture was maintained for 14 days and the medium was changed every other day.

### Immunocytochemistry

All specimens were fixed in 4% paraformaldehyde (Sigma) in phosphate-buffered saline (PBS) for 60 min at room temperature, then washed with PBS 3 times, 5 min each. The specimens were permeabilized with 0.5% Triton X-100 (Sigma) in PBS overnight at 4°C and blocked with 15% basal serum albumin (BSA, Sigma) for 12 h at 4°C. Subsequently, specimens were incubated in primary antibodies diluted in 5% BSA in PBST [0.05% Tween-20 (Sigma) in PBS] overnight at 4°C as follows: mouse monoclonal anti-Nestin (BioLegend,1:500) and mouse monoclonal β-tubulin III (Tuj1; BioLegend, 1:400). After being washed 3 times with PBST, the conjugating secondary antibodies diluted in 5% BSA in PBST were applied for 4 h at room temperature as follows: Cy5, goat anti-mouse (BioLegend, 1:750); and Alexa Fluor 488, goat anti-mouse (Molecular Probes, Eugene, OR, USA, 1:1000). The specimens were then washed with PBST 3 times. Finally, all specimens were mounted and counterstained with ProLong Diamond Antifade Mountant with DAPI (Molecular Probes). Images were taken under BX53 Upright Microscope and FV10i-DOC Confocal Laser Scanning Microscope (Olympus, Alexandra, SG) and processed in ImageJ software and cellSens software (Olympus).

### Evaluation of neurosphere and neuronal differentiation

Neurospheres were monitored and photographed daily under an inverted light microscope (Nikon Eclipse TS100, NIKON INSTRUMENTS, Melville, NY, USA). The quantity and size of the neurospheres were counted and categorized into diameters of 50-150 μm, 151-250 μm, and >250 μm, which were processed in ImageJ software. NSPC was observed by Nestin expression and neuronal differentiation was determined by β-tubulin III expression. The percentage of the expression was calculated considering the total cell number (DAPI staining).

### Statistical analysis

All experiments were done in triplicate. Statistical tests were performed with GraphPad Prism 5 software. The data were expressed as mean ± standard deviation (SD). One-way analysis of variance (ANOVA) was used when comparing differences between groups and Tukey’s test was applied for multiple comparisons. An independent *t*-test was used when comparing the differences between the two treatments. P-values <0.05 were considered statistically significant.

## Results

### Cells isolated from pulp tissue of human permanent teeth show mesenchymal stem cell characteristics.

**Figure 1:**
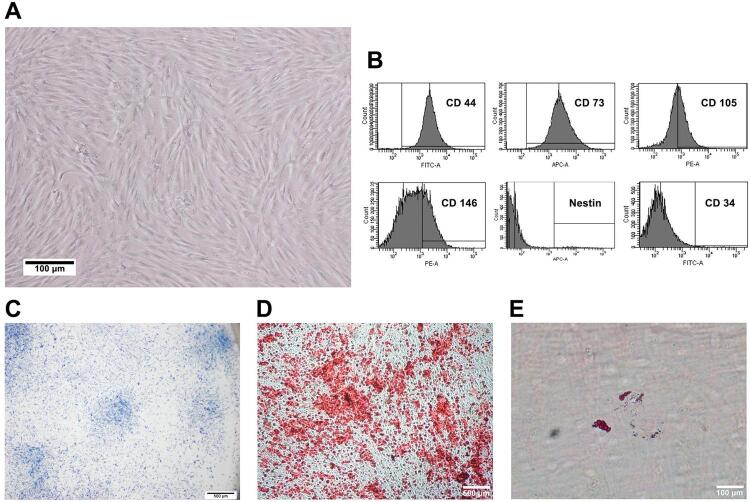
The isolated cells from human permanent teeth exhibited mesenchymal stem cell characteristics and were defined as DPSCs. (A) A light microscopic image of the cells showing fibroblast-like shape. (B) Flow cytometry analysis shows expressions of CD44, CD73, CD105, CD146, Nestin, and CD34 of the cells. (C) Colony-forming unit-fibroblast assay of the isolated cells under a light microscope. (D) Alizarin red staining of osteogenic differentiated cells as indicated by red mineralized nodules. (E) Adipogenic differentiation of the cell observed by oil red O lipid droplets staining

The isolated cells exhibited a spindle-like shape similar to fibroblasts (Figure 1A). The cells highly expressed mesenchymal stem cells markers CD44 (96.93±0.82%) and CD73 (98.83±0.46%), and moderately expressed CD105 (63.97±11.18%), and CD146 (44.00±17.00%). The cells slightly expressed NSPC marker, Nestin (5.47±2.49%), but negatively expressed hematopoietic stem cell marker CD34 (0.63±0.12%) (Figure 1B). The colonies were observed when they were cultured in low density, demonstrating that the cells were capable of self-renewal. (Figure 1C). The cells were differentiated into osteoblast and adipocyte, which is indicated by alizarin red staining of mineralized nodules (Figure 1D) and oil red O staining of lipid droplets (Figure 1E), respectively. Thus, the cells can be defined as DPSCs.

### Neurosphere culture requires culture vessel inhibiting cell attachment

**Figure 2:**
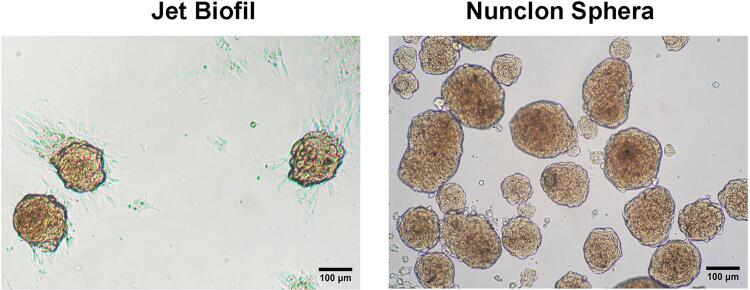
The light microscopic images show the morphology of neurospheres generated from DPSCs. Neurospheres 4-days cultured on the Jet Biofil non-treated plate attached to the surface of the plate (Left). Neurospheres cultured on the Nunclon Sphera plate freely floated and showed smooth rims (Right)

To establish the 3D culture of the free-floating cell cluster, several commercial culture vessels were purported to prevent surface attachment of the cells. Jet Biofil non-treated plates and Nunclon Sphera plates were used to compare the efficiency of the neurosphere assay. After 24 hours of culture in the NSPC medium, the cells clustered together forming numerous neurospheres in both culture plates. However, some cells or cell clusters attached to the Jet Biofil non-treated plate, and only a few neurospheres were able to move freely. Unfortunately, the floating neurospheres were absent by day 4 of the culture. All of the cell clumps and neurospheres had attached to the surface of the plate. In contrast, adherent cells were not observed in cultures on the Nunclon Sphera plates. Neurospheres that formed on this plate type were suspended in the culture medium, had a round shape, and varied in size (Figure 2). Thus, Nunclon Sphera plates are suitable to promote neurosphere formation.

### Optimal time for culturing neurospheres is 5 days

**Figure 3:**
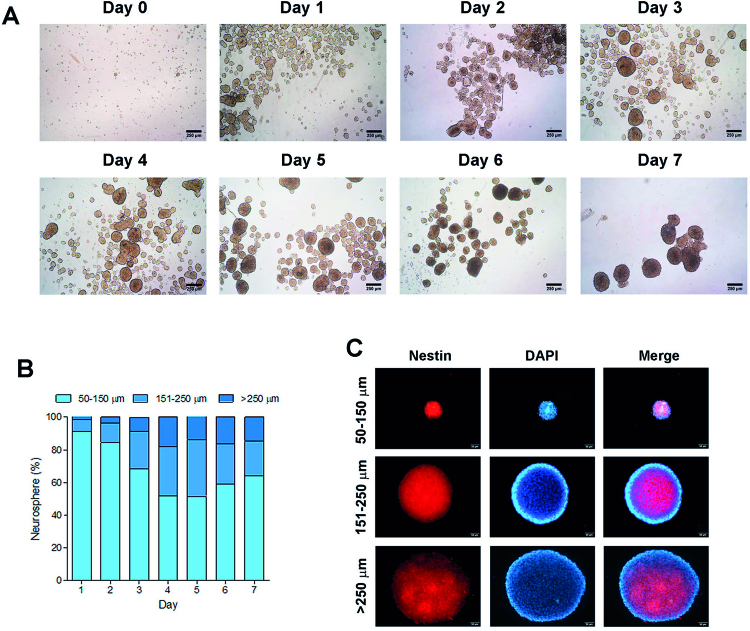
Optimal time for culturing neurospheres. (A) Neurospheres culture from day 0 to day 7. (B) Percentage of each size of the neurospheres on each day of the culture. (C) Immunofluorescence images of representative neurospheres in 50-150 µm, 151-250 µm, and >250 µm of diameter showing the expression of Nestin (red). The nuclei were counterstained with DAPI (blue)

The neurosphere assay was performed and monitored every day of the culture (Figure 3). The neurosphere number decreased over time, whereas the size gradually increased till day 4, and showed several shapes. The neurospheres remained stable size and round shape from day 5 to day 7. The appearance of the neurospheres from day 1 to day 5 of the culture showed homogeneous color, on day 6 they became darkened at their core. By day 7, the dark area expanded and some of the neurospheres had attached to each other (Figure 3A). The size of the neurospheres was classified into diameters of 50-150 µm, 151-250 µm, and more than 250 µm. From the first day of the culture, over 90% of the neurospheres were in the 50-150 um group. This percentage gradually decreased over the following 4 days, then slightly increased again till the end of the culture. The percentage of neurospheres with 151-250 µm diameter increased during the culture, reached their maximum on day 5, and then decreased. The percentage of the >250 µm diameter neurospheres showed a similar trend as the previous groups, increasing from day 1 to day 4, then became constant from day 5 to day 7 (Figure 3B). These results show that 5 days is the most appropriate duration of time to induce DPSCs into NSPCs. Moreover, immunocytochemistry revealed the neurospheres in each size expressed the neural progenitor protein marker Nestin (Figure 3C).

### Neurospheres are well dissociated by Accutase

**Figure 4:**
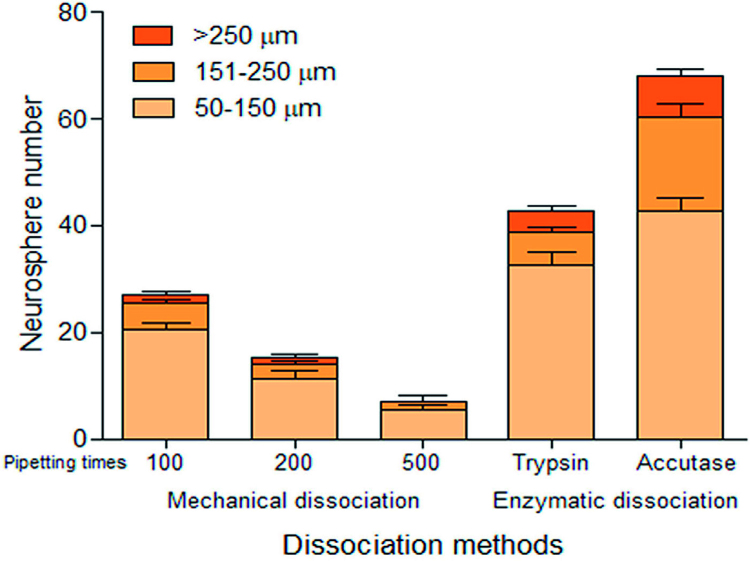
Neurosphere numbers generated from each dissociation method. Mechanical dissociation by repeated pipetting with 100, 200, and 500 times and enzymatic digestion by Trypsin and Accutase were showed neurosphere re-forming numbers in 50-150 µm, 151-250 µm, and >250 µm of diameter

Neurospheres can be subcultured for propagating an amount of NSPC or seeding a 2D culture by dissociation.^24^ In our study, mechanical dissociation by repeated pipetting, with various times and enzymatic digestion by Trypsin and Accutase, were compared for the efficiency of neurosphere re-forming. A 100-time pipetting generated the highest neurosphere number and the efficiency decreased when repeated pipetting times were increased (Figure 4). However, the number of the re-forming neurosphere from the 100-time pipetting was significantly lower compared to enzymatic dissociation. Comparing two enzymes, the neurospheres generated from Accutase disaggregation were significantly higher than Trypsin disaggregation (Figure 4). Therefore, dissociation of neurospheres by Accutase resulted in significantly higher viable neurospheres than trypsin and mechanical dissociation.

### Passage number does not affect NSPC induction but reflects neuronal differentiation outcomes

**Figure 5:**
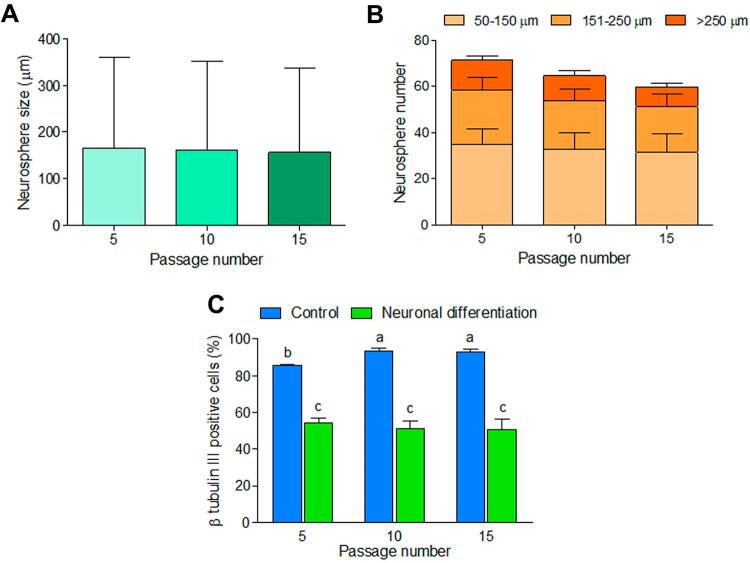
Effect of passage number of DPSCs on neurosphere formation and neuronal differentiation. (A) Average size, (B) average number, and (C) percentage of β tubulin III-expressing cells derived from DPSCs at passage 5, 10, and 15 after cultured the cells in neuronal differentiation medium compared to control medium. Bars with different alphabets are significantly different (p<0.05)

DPSCs at passage 5, passage 10, and passage 15 could form neurospheres without significant differences in average size (Figure 5A) and number (Figure 5B) of neurospheres. However, neurospheres size and number derived from DPSCs at passage 5 (166.28 and 71.44) was trended higher compared with passage 10 (162.18 and 64.78) and passage 15 (156.03 and 60.00), respectively. NSPCs derived from DPSCs at passage 5, 10, and 15 in the neuronal differentiation group showed a comparable percentage of β-tubulin III expressing cells (passage 5: 54.08±2.70%; passage 10: 51.12±4.15%; passage 15: 50.90±5.52%), whereas the expression of the cells at passage 5 in the control group was significantly lower than passage 10 and 15 (passage 5: 85.57±0.66%; passage 10: 93.22±1.91%; passage 15: 93.11±1.23%) (Figure 5C). Surprisingly, the percentage of β-tubulin III expressing cells in the control group was significantly higher than the neuronal differentiation group when comparing the same passage between the groups.

**Figure 6:**
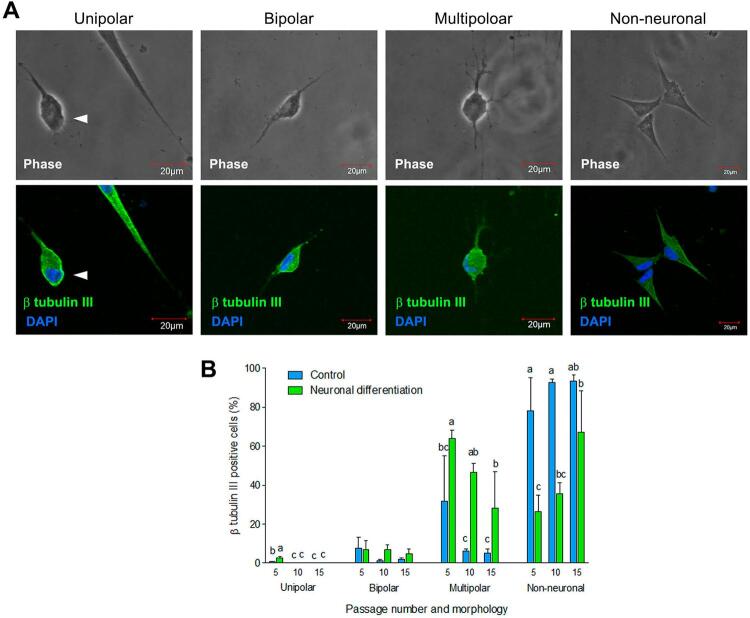
Effect of passage number of DPSCs on neuronal morphological change after cultured the cells in neuronal differentiation medium compared to control medium. (A) Unipolar (arrowhead), bipolar, multipolar, and non-neuronal morphology observed in the β-III tubulin-expressing cells. The upper panel is phase contrast images and the lower panel is immunofluorescence images; green: β-tubulin III; blue: DAPI. (B) Percentage of β-tubulin III-expressing cells that exhibit each type of neuronal morphology. Bars with different alphabets are significantly different (p<0.05)

Moreover, Figure 6A shows the examination of β-tubulin III expressing cells for neuron characteristics by classifying their neuronal morphology into 4 groups: unipolar morphology (arrowhead), a round-shaped cell body with a single process; bipolar morphology, a round-shaped cell body with two slender processes; multipolar morphology, a relatively round-shaped cell body with multiple processes; and non-neuronal morphology, a fibroblast-like or spindle-shaped cell. Regarding passage number in the control group, the percentage of cells from passage 5 with unipolar morphology was significantly higher than the cells from passage 10 and 15 (Figure 6B). The percentage of cells with 3 remaining morphologies was not statistically different amongst the 3 passages. In the neuronal differentiation group, the percentage of cells with unipolar morphology from DPSCs passage 5 was significantly higher than those of passage 10 and 15 (Figure 6B). The percentage of cells with bipolar morphology was not significantly different among the 3 passages. The percentage of multipolar cells from passage 5 was significantly higher than those of passage 15. The cells at passage 5 with non-neuronal morphology showed a statistically lower percentage than the cells at passage 15 (Figure 6B). When comparing neuronal differentiation and control groups for the percentage of each kind of neuronal morphology, we found that the percentage of cells with unipolar morphology in the neuronal differentiation group was significantly higher than the control group at passage 5. The bipolar cells in the neuronal differentiation group showed a comparable percentage as the control group in all passages. The percentage of cells with multipolar morphology in the neuronal differentiation group was significantly higher than the control group. For the non-neuronal cells, the percentage of cells at passage 5 and 10 in the control group was statistically higher than the neuronal differentiation group, but the cells at passage 15 were not statistically different (Figure 6B).

## Discussion

Neuronal differentiation of DPSCs has been extensively studied for over a decade for the purposes of neurodegenerative disease treatment, modeling, and understanding developmental biology. Methods of inducing differentiation are numerous and differ according to the target neuron types, which require different induction cues. DPSCs are committed into neural lineage by neurosphere assay to enable the expression of neuron-associated molecules and prevent differentiation into undesired lineages. They are then terminally differentiated into the target neurons, or transplanted *in vivo*.^25^ However, most existing studies overlooked an evaluation of the neurosphere formation that resulted in varied protocols for producing the neurospheres. Enhancement of neurosphere formation by adjusting optimal conditions can maximize NSPC generation and potentially contribute to a higher yield of neurons. In our study, we optimized the culture conditions that promote neurosphere formations of high quality and yield by varying culture vessels, duration of cultures, and dissociation methods. Passage number was also investigated for neurosphere generation and extended to neuronal differentiation. The efficiency of each condition was determined in reference to neurosphere number, size, and Nestin expression. The size of the human neurosphere directly reflects the proliferative potential of NSPCs as it increases along with ATP production.^26^ Nestin is a class VI intermediate filament protein found exclusively in neuroepithelial stem cells that later give rise to neurons and glias.^27^ The expression of Nestin in NSPCs is downregulated once the cells differentiate.^28^ The spatiotemporal expression of Nestin makes it an excellent marker to identify NSPCs, and has been used extensively.

The culture surface materials that support cellular events and resemble the biological niche of the tissue deserve especial attention.^29^ One challenge of neurosphere generation is the facilitation of unattached cell aggregation, since 3D neural induction increased NSPC markers and generated longer neurites compared to 2D induction.^30^ Chemical modification of the surface of culture vessels can prevent cell attachment and promote the cell division that forms the neurospheres. We compared two culture plates from different companies. Jet Biofil non-treated plates are less expensive and have been used for neural stem cell cultures in a previous study.^31^ However, the seeded cells attached to the plate and were unable to form neurospheres due to a hydrophobic surface. Unlike non-treated plates, free-floating spherical aggregates of cells were present when using Nunclon Sphera plates, without any cells attaching to the plate surfaces. The hydrophilic polymer-coated surface of the Nunclon Sphera plate inhibits protein absorption, thus preventing cell attachment.^32^ The most variable is the duration of the neurosphere culture. We monitored the neurospheres every day of the 7-day culture period, day 5 of neurosphere culture was the optimum time which the typical culture duration in most previous studies.^12,21,33^ The number of neurospheres decreased over time within the cultures while their size increased, corroborating a previous study that demonstrated that neurospheres are generated from self-renewal and sphere aggregation.^26^ Neurospheres were categorized into 3 groups according to size. The neurospheres with less than 250 µm diameter were translucent, showed regular rims and well-defined spherical shapes, which are considered to be characteristics of healthy neurospheres.^34^ We focused on the neurospheres with 150-250 µm diameter, since they represented the highest proliferation of NSPCs. The proportion of this size of neurosphere was highest on days 4 and 5, with day 5 showing slightly higher than day 4. When the neurospheres reached 250 µm of diameter, the ATP production is highest and remains unchanged as the neurosphere grow bigger.^26^ Moreover, they exhibited a dark color, as observed in many neurospheres on day 7 of the culture, indicating that the cells were oxygen and nutrient deprived and also unable to eliminate metabolites. Metabolic activity and the percentage of cells in the G0/G1 of neurospheres on day 5 and day 7 of the culture are not significantly different.^12^

Dissociation of the neurosphere is needed for the amplification of the NSPC population and neuronal differentiation. It is a critical process to preserve the viability of the NSPCs. Mechanical dissociation by repeated pipetting is convenient and cheap, since it requires equipment that is already available for routine lab work. Mechanical dissociation generates traumatic force that damage cells, resulting in an over 40% reduction of viable cells.^35^ Trypsin is a protease commonly used to digest cell adhesion molecules to detach cells from plastic ware. Accutase, a gentler enzyme, has been developed to replace trypsin and is useful for flow cytometry analysis, since it preserves most cell epitopes (http://www.accutase.com/accutase.html). Accutase is understood to preserve more surface molecules, such as receptors of growth factors. As a result, cells are more responsive to EGF and bFGF, and are more viable than cells dissociated with trypsin.^33^ The study showed that Accutase is an effective enzyme for dissociation of DPSCs-derived neurospheres.

To obtain sufficient stem cell numbers for study or therapeutic use, the cells must be subcultured several times. Increasing the passage number via subculture is *in vitro* aging, which changes mesenchymal stem cell properties including abnormal morphology, inhomogeneous population, and loss of differentiation potential.^36^ Surprisingly, our results showed that 3 passages; 5, 10, and 15, of DPSCs exhibited comparable results for neurosphere formation. Cell proliferation of DPSCs at passage 2 and 15 remain unchanged; however, neurogenic markers such as NEUROG1, REST, and ZFHX3 increase their expression at passage 15, suggesting that DPSCs commit themselves into a neural lineage in the late passages.^37^ Neuronal differentiation is determined by β-tubulin III expression, a protein expressed specifically in neurons.^38^ DPSCs-derived NSPCs in the neuronal differentiation group showed comparable percentages of the marker expression among different passages, but lower than the control group. When NSPCs are changed from a neurosphere culture into an adherent culture, they stop proliferation and become differentiated,^7^ resulting in high expressions of the neuronal markers. Moreover, the neurogenic properties of DPSCs could make them spontaneously differentiate into neuron-like cells without neural induction. We then evaluated the neuronal morphology of the β- tubulin III-expressing cells to find other differences between the control and neuronal differentiation groups. The distribution of each type of neuronal morphology in our results were typical of those found in vertebrates, since the most common neurons are multipolar.^39^ As we expected, the β-tubulin III-expressing cells in the neuronal differentiation group showed significantly greater neuronal morphologies than the control group in early passages. Neurotrophins added in the neuronal differentiation medium alter the dynamics of the cytoskeleton and promote neuronal maturation of the NSPCs.^40,41^ However, the percentage of cells with neuronal morphologies declined when the passage number increased. A previous study reported that mesenchymal stem cells from human bone marrow upregulate expression of GFAP, a glial cell marker, in the late passages.^42^ This report could explain our results that some cells in the late passage possibly inclined toward glias.

## Conclusions

In our study, we propose a guideline for neurosphere generation from DPSCs. The culture requires modified vessel surfaces to inhibit cellular attachment and promote 3D spheroid formation. A 5-day culture duration is an optimal time allowing high proliferative NSPCs to form neurospheres. Dissociation of the neurospheres should be done using Accutase digestion to obtain a high number of viable cells. Finally, to differentiate or transplant NSPCs derived from DPSCs, we suggested inducing the neurosphere from early passage DPSCs to maximize the neuronal differentiation. However, to use the cells for therapeutic applications, the NPSCs obtained from this protocol require more study on their characteristics. Further *in vivo* studies should be conducted to test the efficacy of DPSC-derived NSPCs in the recovery of the functional nervous system. This preclinical data would offer information to develop clinical NSPCs from DPSCs that benefits both allotransplantation and autologous transplantation.
